# Fast reconstruction of milling temperature field based on CNN-GRU machine learning models

**DOI:** 10.3389/fnbot.2024.1448482

**Published:** 2024-09-27

**Authors:** Fengyuan Ma, Haoyu Wang, Mingfeng E, Zhongjin Sha, Xingshu Wang, Yunxian Cui, Junwei Yin

**Affiliations:** ^1^School of Mechanical Engineering, Dalian Jiaotong University, Dalian, China; ^2^Angang Heavy Machinery Co., Ltd, Anshan, China

**Keywords:** temperature field reconstruction, gated convolutional neural networks, knowledge distillation, inverse heat transfer, milling

## Abstract

With the development of intelligent manufacturing technology, robots have become more widespread in the field of milling processing. When milling difficult-to-machine alloy materials, the localized high temperature and large temperature gradient at the front face of the tool lead to shortened tool life and poor machining quality. The existing temperature field reconstruction methods have many assumptions, large arithmetic volume and long solution time. In this paper, an inverse heat conduction problem solution model based on Gated Convolutional Recurrent Neural Network (CNN-GRU) is proposed for reconstructing the temperature field of the tool during milling. In order to ensure the speed and accuracy of the reconstruction, we propose to utilize the inverse heat conduction problem solution model constructed by knowledge distillation (KD) and compression acceleration, which achieves a significant reduction of the training time with a small loss of optimality and ensures the accuracy and efficiency of the prediction model. With different levels of random noise added to the model input data, CNN-GRU + KD is noise-resistant and still shows good robustness and stability under noisy data. The temperature field reconstruction of the milling tool is carried out for three different working conditions, and the curve fitting excellence under the three conditions is 0.97 at the highest, and the root mean square error is 1.43°C at the minimum, respectively, and the experimental results show that the model is feasible and effective in carrying out the temperature field reconstruction of the milling tool and is of great significance in improving the accuracy of the milling machining robot.

## 1 Introduction

In the era of Industry 4.0, China’s manufacturing industry is undergoing a profound transformation, and the use of robotics is becoming increasingly important in intelligent manufacturing. Intelligent manufacturing relies on multifunctional sensors to perceive the production environment (Cheng et al., 2016; Javaid et al., 2021). Production equipment autonomously learns through sensor-based and data-driven methods. This enables adaptive machining in changing environments. Ultimately, intelligent control achieves the desired outcomes (Lee et al., 2015). As one of the important machining methods in the manufacturing industry, milling processing has a broad prospect for the use of robots. In the use of robots, the most important issue is the processing quality and processing accuracy. In milling machining, localized high temperatures and strong time-varying temperature gradients are mainly concentrated at the boundary of the tool heat transfer system, i.e., the cutting region. Localized high temperatures impact tool life and can stimulate the chemical activity of the material being removed. This leads to material oxidation, rapid corrosion, and adhesion and diffusion between the material and the tool. Consequently, these effects degrade the machining accuracy and quality of the workpiece (Korkmaz and Gupta, 2024). In addition, the high temperature of the cutting area will also cause localized thermal deformation of the tool tip, which is one of the main reasons for the reduction of machining accuracy. Therefore, the localized high temperature in the cutting area and the strong time-varying temperature gradient will lead to the shortening of tool life, the reduction of workpiece machining quality, and the reduction of machining efficiency.

Due to the interference of cutting fluid and chips, existing sensing technology cannot directly measure the temperature field inside the cutting area (Alammari et al., 2024). Sensors can only be placed near the tool-chip contact area to obtain limited temperature data outside the cutting zone. Metal cutting is a thermodynamic coupling process with significant changes in material elastic–plastic deformation and contact area friction. These changes cause strong non-uniformity in the tool temperature field over time and space. A single or a few measurement points cannot accurately describe the actual processing conditions. Therefore, studying tool temperature field reconstruction during milling is crucial for extending tool life and improving machining accuracy.

Currently, there is still some difficulty in accurately measuring the temperature field online for the cutting region, and physical methods based on infrared thermography, artificial thermocouples and embedded thermocouples (Cichosz et al., 2023; Leonidas et al., 2022; Longbottom and Lanham, 2005) can only measure a limited number of *in-situ* temperatures near the cutting region or the approximate temperature field close to the location of the cutting region. In recent years, computational reconstruction methods for modeling the temperature field of tools have gained widespread attention. These methods bypass physical limitations to obtain temperature data at any location of interest. Current modeling techniques are mainly categorized into analytical modeling, numerical simulation based on cutting mechanisms, and inverse heat conduction modeling, which combines physical measurements with model-solving methods. The inverse heat conduction problem (IHCP) is part of the “mathematical physics inverse problem” field. A positive problem in physics research can be described by mathematical equations, where given equations and parameters, the output can be determined from a known input. Early studies simplified tool models and the cutting process, often treating the tool as a one- or two-dimensional model and the cutting process as steady-state. In these cases, analytical methods were combined with IHCP to directly compute mathematical expressions for the relationship between unknown quantities and measured values (Murio, 1981).

Nowadays, more and more researchers are focusing on reducing the complex three-dimensional structure of the tool with transient cutting process (Oommen and Srinivasan, 2022), for example, Some scholars (Liang et al., 2013) proposed a three-dimensional inverse heat transfer model based on an improved conjugate gradient method, which can quantitatively calculate the temperature of the tool chip contact area in dry turning. Some other researchers (Carvalho et al., 2006) used the golden section iterative method to solve the inverse heat conduction problem, and used the finite volume method to construct a three-dimensional model of the turning tool, which takes into account the thermal properties of the material as affected by temperature as well as the convective heat transfer losses to realize the temperature field reconstruction calculation.

In recent years, with the advancement of artificial intelligence algorithms and machine learning technology, artificial neural network models based on data relations have been widely used in inverse thermal problem solving. For example, the application of algorithms such as physical information neural network (PINN; Qian et al., 2023), nonlinear autoregressive exogenous input neural network (NARX; Chen and Pan, 2023), convolutional neural network (CNN; Kim and Lee, 2020), and multidomain physical information neural network (M-PINN; Zhang et al., 2022), etc., has made a certain contribution to the solution of the inverse heat conduction problem. Researchers (Zhang and Wang, 2024) have used deep neural networks to characterize and approximate partial differential equations (PDEs) in the forward problem style. They proposed an optimization algorithm that uses sequence-to-sequence (Seq2Seq) stacking with the gated recurrent unit (GRU) model. It improves the solving of these equations by stacking GRU modules to capture their evolution over time. It also has strong generalization ability.

There is still a wide range of prospects for the fusion of artificial neural network models. For example, CNN struggles to capture temporal features, while GRU struggles to capture spatial features. Combining CNN and GRU might allow their strengths to complement each other, enabling the CNN-GRU model to effectively capture spatio-temporal features, thereby improving the model’s accuracy and generalization performance.

This paper proposes a CNN-GRU based milling tool heat transfer model with knowledge distillation compression acceleration. The model reconstructs the milling tool temperature field under three different working conditions. A self-built milling temperature data acquisition system collects real-time temperature data from multiple points on the back face of the milling cutter. This system uses a temperature measurement tool embedded with a thin-film thermocouple array and a multi-channel signal acquisition device. By analyzing the relationship between machining parameters, the temperature at four measurement points on the milling tool, and the temperature in the cutting area, we use machining parameters and multi-point temperatures as input features. The temperature boundary conditions in the cutting area serve as prediction labels. The GRU is introduced to the convolutional neural network (CNN) to extract multi-dimensional feature information, aiming to improve reconstruction accuracy and efficiency. We then apply a knowledge distillation strategy to compress and accelerate the CNN-GRU model. This approach reduces computation time while maintaining high prediction performance and accuracy, ensuring efficient temperature field reconstruction.

The rest of this study is organized as follows: section 2 reviews the work related to this study, section 3 describes the proposed method in detail, section 4 reports the experimental results and analysis, and section 5 concludes this study.

The contributions of this paper are as follows:

1. CNN-GRU-based solution model for inverse heat transfer problem: a solution model for inverse heat transfer problem based on convolutional gated recurrent network (CNN-GRU) to predict the temperature boundary conditions in the cutting region of the tool is proposed. The model can well utilize the machining parameters in milling processing, the characteristics of the multi-point temperature of the milling tool back face, thus significantly improving the accuracy and efficiency of the milling temperature field reconstruction.
2. KD compression-accelerated model for solving inverse heat conduction problem: the constructed CNN-GRU model is compressed and accelerated using the knowledge distillation strategy. Compared with the model without KD acceleration, the model can substantially accelerate the training time with the least loss of goodness-of-fit, and has strong noise immunity.
3. A transient heat conduction model of milling tool is constructed, and the temperature field reconstruction of milling tool is carried out for three different working conditions, and the tests under the three working conditions are carried out in order to check its application ability in the reconstruction of milling temperature field.

## 2 Related work

Currently, deep learning techniques have been successfully applied to real-world scenarios, solving challenging problems like predicting the lifetime of relays and batteries. Constructing prediction models with artificial neural networks has broad applications in solving inverse heat transfer problems (Cortés et al., 2007; Wang et al., 2023; Kamyab et al., 2022). Additionally, methods for compressing and accelerating deep learning models have enhanced the efficiency and applicability of these techniques in real-time temperature field reconstruction. Integrating these advanced algorithms significantly improves the precision and speed of temperature field predictions during milling. This makes them indispensable for optimizing machining operations.

### 2.1 Shallow artificial neural network approach

Shallow artificial neural network methods were among the first techniques applied to the solution of inverse heat transfer problems. These methods utilize a simple hierarchical structure for data processing and prediction by simulating the way neurons in the brain work. Despite the simplicity of their structure, shallow neural networks have demonstrated their effectiveness and feasibility in solving specific problems, such as in the area of predicting lifespan.

Combining Back Propagation Neural Networks (BPNNs) with time-series data analysis methods has been utilized to predict the remaining life of cooling fans (Lixin et al., 2016). Based on the time series data analysis of historical data information to obtain the future trend of the data, the prediction error is adjusted using BPNN to ensure the accuracy of the prediction results. A single BPNN will face the problem of weight local optimization, i.e., overfitting, during training, and in recent years a large number of scholars have combined BPNN with other machine learning methods to improve the model accuracy.

Radial Basis Function Networks (RBFNs) are widely used in various fields due to their advantages of having outputs independent of initial weights and shorter training times. A gray RBFN-based prediction model (Li et al., 2009) for life and reliability of constant stress accelerated life testing has been developed and compared with traditional single Backpropagation Neural Networks (BPNNs). Experimental results demonstrate that the accuracy of the gray RBFN model surpasses that of the BPNN.

The shallow artificial neural network model has a high dependence on large-scale data, and the shallow model is prone to overfitting phenomenon during the training process, especially when the training data is small or the data dimension is high. In practice, the sensitivity of shallow artificial neural networks to data quality and noise may lead to a decrease in the robustness of the model.

### 2.2 Deep artificial neural network methods

With the improvement of computational power and the development of deep learning technology, deep artificial neural network methods have demonstrated powerful performance in solving complex problems. Deep neural networks are able to better capture complex patterns and higher-order features in the data through multilayer nonlinear transformations, which significantly improves the predictive ability of the model.

A deep learning method combining sparse stacked self-encoders (Stacked Sparse AEs, SSAEs) with Backpropagation Neural Networks (BPNNs) has been proposed (He et al., 2021). This method uses tool temperature measurements from temperature sensors to predict tool wear. When compared to BPNN and SVM models that rely on manually extracted time-frequency domain features, this approach demonstrates high prediction accuracy and stability.

A time window method for obtaining samples and a multivariate equipment life prediction method based on deep Convolutional Neural Networks (CNNs) have been proposed (Li et al., 2018), focusing on feature extraction. To avoid filtering out effective information by the pooling layer, the pooling layer was removed when constructing the network model. Additionally, a deep CNN method for bearing residual life prediction has been introduced (Ren et al., 2018), which combines spectral principal energy vectors into a feature map. This method extracts one-dimensional vectors and inputs them into the deep learning model through a multilayer CNN structure, demonstrating that its prediction accuracy meets the required standards.

Furthermore, the problem of predicting the remaining life of batteries using deep learning has been explored (Zhang Y. et al., 2018). Long Short-Term Memory (LSTM) networks are used to learn the long-term dependencies between the capacity degradation of lithium-ion batteries. LSTM employs backward error propagation for adaptive optimization and uses the dropout regularization technique to address the overfitting problem. This method exhibits better learning and generalization abilities compared to support vector machines and traditional recurrent neural networks.

The deep artificial neural network method has high accuracy for prediction problems such as lifetime prediction, but there is still a lot of room for improvement in efficiency, and there are some limitations in the application of inverse heat conduction solving problems and temperature field reconstruction.

### 2.3 Deep learning model compression and acceleration method

Neural network pruning is an important method to achieve network model compression and acceleration, and its working principle is mainly to cut off the weights and model branches that are not important when the neural network is working, to get a small model, from achieving the compression and acceleration of the model.

The ThiNet pruning method (Luo et al., 2017) differs from traditional pruning methods by treating network pruning as a reconstruction optimization problem. This approach determines the pruning strategy for the convolutional kernel of the current layer based on statistical information computed from the reconstruction differences between the inputs and outputs of the subsequent layer.

In addition to network pruning, other lightweight network design methods have been developed. Group point-by-point convolution (Zhang X. et al., 2018) performs grouped convolution operations to reduce the computational loss associated with point-by-point convolution operations. To enable grouped convolution to capture features computed by other groups, a mixing operation is introduced to reintegrate features from different groups, allowing the new group to contain features from other groups as well.

Automatic machine learning algorithms (AutoML) have also been widely used in lightweight neural network design. Some researchers (He et al., 2018) proposed AutoML for Model Compression (AMC), which utilizes reinforcement learning to efficiently sample the design space and learn compression strategies with better compression ratios to maintain model performance while reducing human intervention in the model. and maintain model performance while reducing human intervention in the model.

Due to the large model capacity difference between the teacher model and the student model, which leads to a “generation gap” between the student model and the teacher model, Wang Y. et al. (2018) pioneered a teacher-assistant-assisted knowledge distillation method, which utilizes the discriminator of the generative adversarial network as the teacher-assistant. They regarded the student model as a generator, and guided by the discriminator, the student model generated a feature distribution similar to that of the teacher’s model, thus assisting the student model in learning. Some researchers (Cui et al., 2017) proposed a novel mutual distillation method, which allowed two groups of untrained student models to start learning and solve the task together, i.e., the teacher and the student models were trained and updated at the same time.

According to the above findings, knowledge distillation, a deep learning model compression and acceleration strategy, has been widely applied and developed, but little research has been reported on the application of knowledge distillation techniques in the field of heat conduction inverse problem solving.

### 2.4 Temperature field reconstruction

Temperature field reconstruction is a key step in solving inverse heat transfer problems, through which accurate reconstruction of the temperature field can lead to a better understanding of the heat transfer process and improve the thermal performance of materials and devices. In recent years, temperature field reconstruction techniques combining advanced algorithms and neural network methods have made significant progress.

An enhanced Bayesian backpropagation neural network based on Kalman filtering has been proposed (Deng and Hwang, 2007), applying the Kalman filtering algorithm to improve the weak generalization ability of the backpropagation algorithm in approximating nonlinear functions. This enhancement improves the performance of the Bayesian backpropagation network in solving the inverse heat conduction problem, and it has been compared with backpropagation networks optimized using other mature algorithms, such as GMB and LMB.

In another study, the volumetric heat capacity function of solid materials with temperature has been solved using a backpropagation neural network combined with a radial basis function neural network based on full-history information (Czél et al., 2013). Some researchers (Wang H. et al., 2018) proposed a heat flux estimation algorithm based on a linear artificial neural network for identifying a finite shock response under a linear dynamic system.

In conclusion, temperature field reconstruction plays an important role in the solution of inverse heat conduction problems. By introducing neural networks and other intelligent algorithms, researchers have made many breakthroughs in improving the reconstruction accuracy and computational efficiency. These methods not only enrich the means of solving inverse problems theoretically, but also demonstrate a strong potential in practical applications, providing new ideas for the solution of complex heat conduction problems.

## 3 Methods

### 3.1 Acquisition of data sets

**Figure 1:**
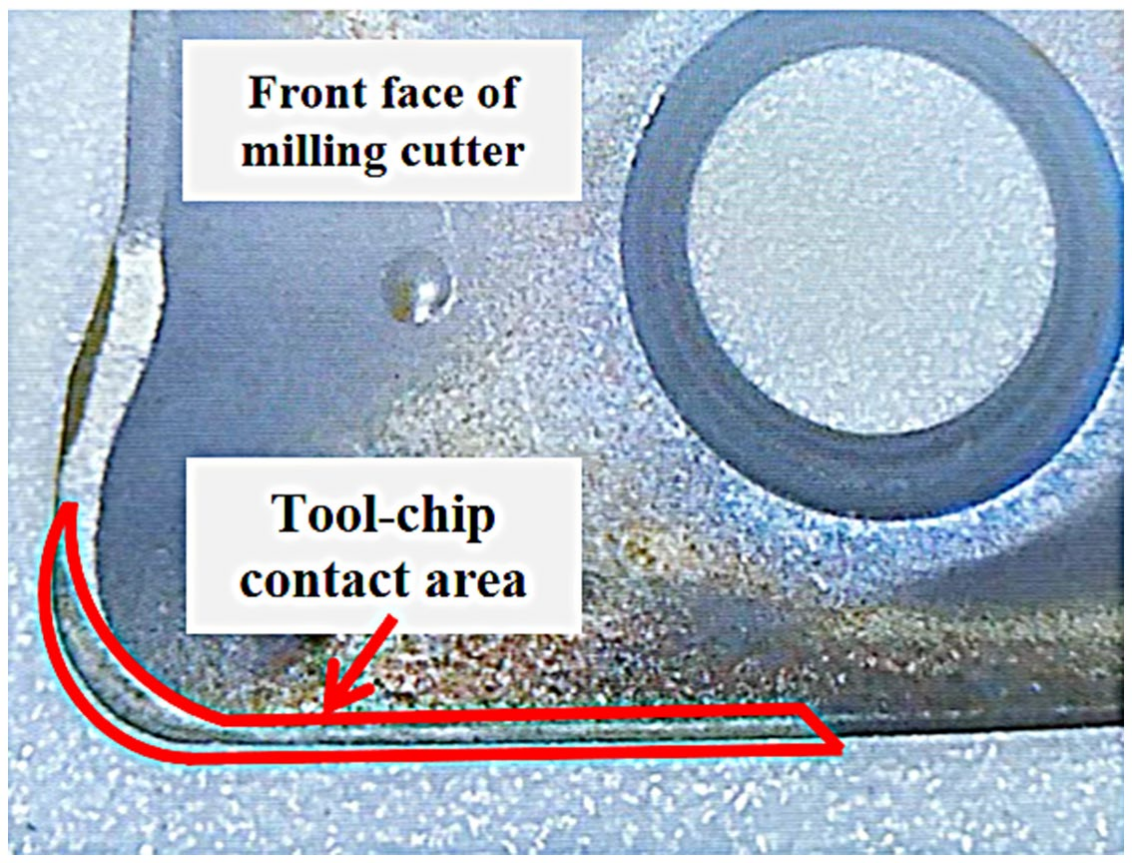
Image of the cutting area of the tool with radial depth of cut (ae) of 0.2 mm and axial depth of cut (ap) of 8 mm.

In metal cutting, the temperature of the tool is mainly affected by the integrated heat source of the three deformation zones, in which the heat is mainly transferred to the tool through the cutting area, and the cutting area of the tool can be regarded as the boundary of the tool heat conduction system. The cutting region of the tool generally includes: the tool-chip contact region and the tool-worker contact region, when the tool back angle is large, the cutting time is short and the cutting speed is small, and the back face of the tool does not undergo intense wear, the tool-worker contact region can be regarded as a part of the tool-chip contact region (Jaspers et al., 1998), and at this time, the tool-chip contact region is the tool’s cutting region. The front face of the tool can be photographed using an electron microscope, and the wear area of the main cutting edge attachment is the tool-chip contact area. Figure 1 shows the image of the cutting area of the tool with radial depth of cut (ae) of 0.2 mm and axial depth of cut (ap) of 8 mm.

**Figure 2:**
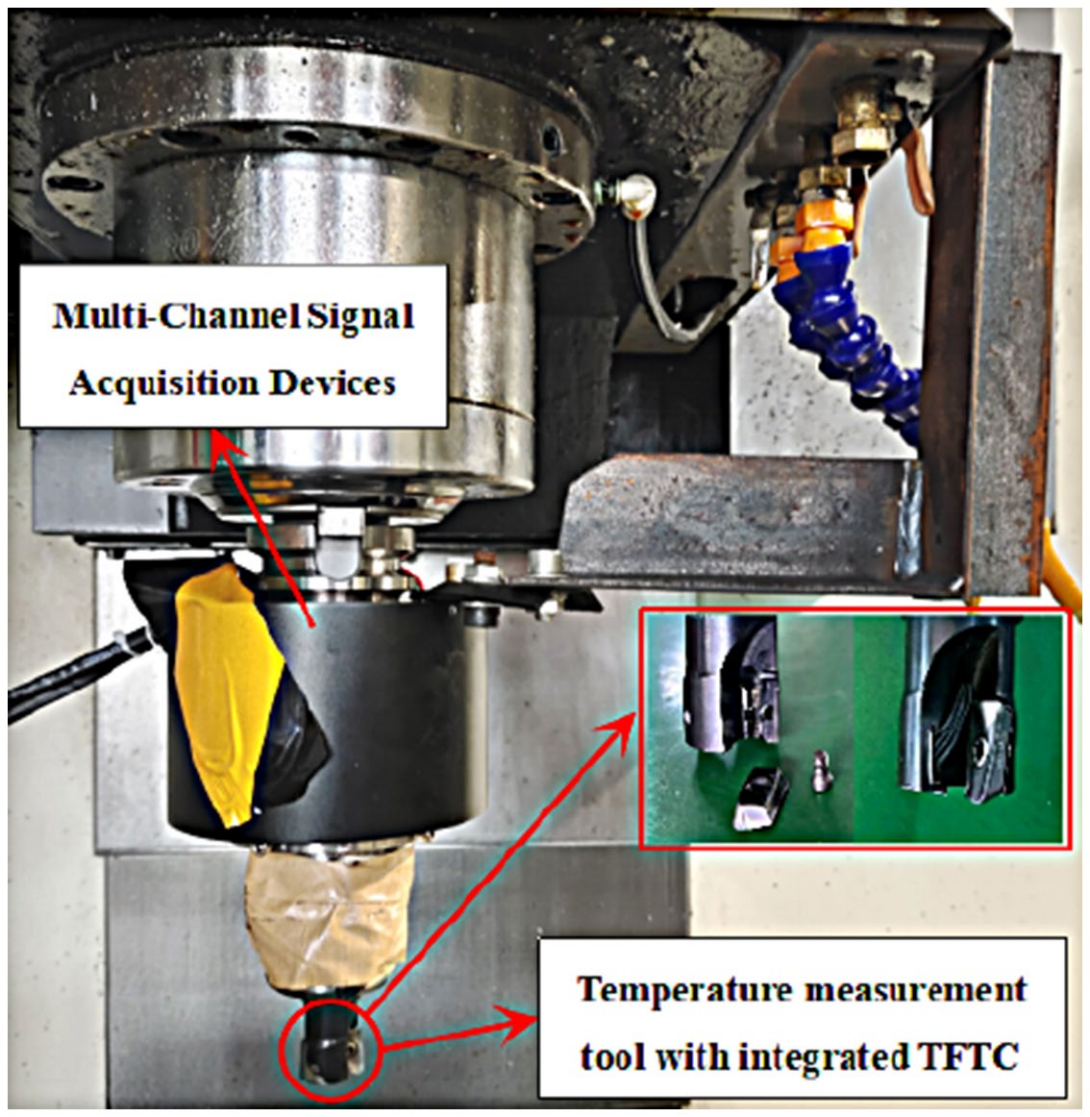
Milling multi-point temperature measurement toolholder.

In this test, a temperature measuring tool embedded with a thin-film thermocouple (TFTC) developed by this group was used for end milling Inconel 718 nickel-based high-temperature alloy workpiece, and according to the requirements of the test, the size of the workpiece was designed to be 50 mm × 20 mm × 10 mm. In the design of the test for end milling Inconel 718, the comprehensive consideration of the theory of heat transfer of metal cutting was taken into account, and the spindle speed (r/min), feed rate (mm/min), and radial milling depth (mm), which have an important influence on milling temperature, were taken as test variable factors. The spindle speed (r/min), feed rate (mm/min), and radial milling depth (mm), which have an important influence on the milling temperature, are taken as the test variable factors. After determining the test variables, a full factorial design of experiments (DOE) was used to ensure that all levels of each test variable were tested at least once. Figure 2 shows a physical diagram of a transient milling multi-point temperature measurement toolholder.

The end milling test was conducted using the constructed test platform and test program, and the temperature data corresponding to the four temperature measurement points of the milling tool were recorded and saved. According to the actual processing requirements, when reconstructing the temperature field of the milling process tool, only the temperature field reconstruction of the tool during the cutting process needs to be considered, without the need to reconstruct the temperature field of the retracting process after the completion of machining, so this paper in the subsequent processing of data in the process of selecting the cut in to the cut out of the retracting tool before the start of the temperature reduction moment to be recorded. In the end milling process, the tool is often accompanied by violent vibration, so the collected temperature data will have a certain noise level, and the data need to be filtered.

The inverse heat conduction problem of the tool heat conduction model refers to the fact that one of the parameters in the control equations, initial conditions, thermophysical parameters, and all boundary conditions of the tool heat conduction is in a missing state, and the unknown parameters need to be solved in reverse by measuring the physical signals by other methods. The inverse heat conduction problem in this paper belongs to the first type of margin estimation inverse problem, where the temperature on the boundary of the tool heat conduction system is estimated from the temperature sensor measurement results. The temperature on the boundary of the tool heat transfer system cannot be measured by physical methods or the measurement accuracy is poor, and is generally obtained using simulation methods. By constructing a local numerical simulation model of the milling process, the Inconel 718 end milling simulation model is operated and set up in complete control of the full factorial test parameters and machining time, and the simulation model is adjusted and corrected by the results of the actual sensor measurements and comparison of the chip morphology. The test simulation was completed using a cutting model with the required accuracy to obtain temperature data on the boundary of the tool heat transfer system.

The simulation model is adjusted and calibrated according to the test results, and after the accuracy meets the requirements, the average temperature of the cutting area of the tool is derived from the cloud diagram of the simulation results, and the other 26 sets of temperature curves can also be obtained by the simulation model, which provides the data sample set of the training model for the subsequent inverse heat conduction problem solving.

### 3.2 Construction of gated convolutional recurrent network model

The traditional one-dimensional CNN may ignore the time series features in the input data, resulting in the loss of some important time series information, in order to solve this problem, GRU can be introduced on the basis of one-dimensional CNN to simultaneously extract the multi-dimensional feature information as well as the temporal characteristics of the time series. Gated Convolutional Recurrent Neural Network (CNN-GRU) is a kind of neural network that combines the features of both CNN and GRU models, and is usually used to process time-series data, text, speech, and video, etc. The workflow of CNN-GRU is firstly, the input data undergoes a series of Convolution and pooling operations to extract the spatial dimension information in the data, and then the local features after the convolution operation are input into the GRU for sequence modeling, the GRU will dynamically update the hidden state according to the feature sequences of the input data, obtaining the long-term dependencies in the input data, and perform the task of output prediction according to the hidden state of the GRU network, and the final output is performed through a fully connected layer. Using CNN-GRU as a model for solving the inverse heat transfer problem can directly establish the nonlinear data relationship between the machining parameters, the temperature of the back face of the milling cutter and the temperature of the cutting area. The CNN effectively captures the spatial information and combines with the GRU network to model the long-term dependence in the sequence to realize the rapid solution of the nonlinear inverse heat transfer problem.

**Figure 3:**
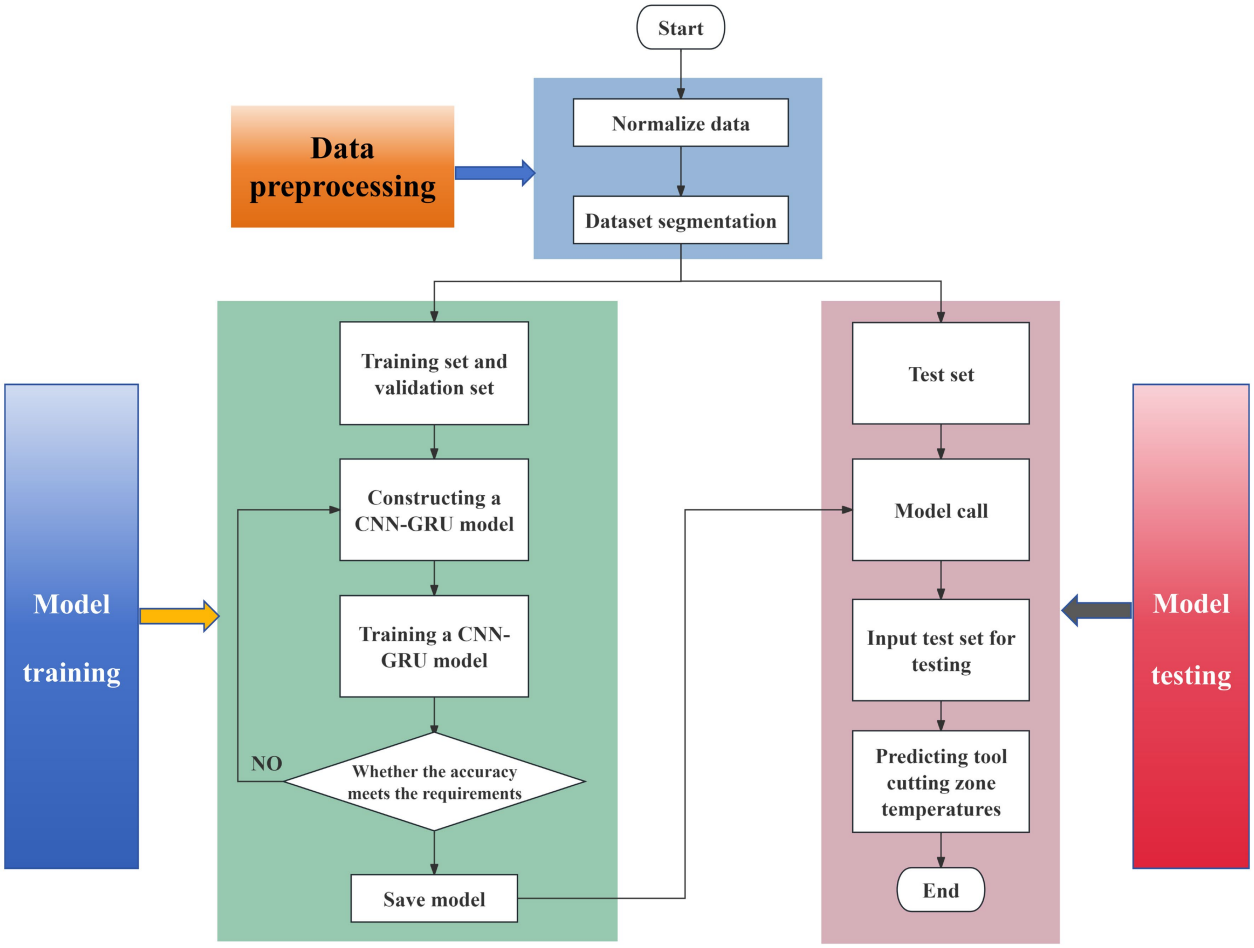
Prediction process based on the CNN-GRU model.

The prediction process based on the CNN-GRU model is shown in Figure 3 as follows:

1. Preprocess the original dataset with data normalization and dataset division;
2. Construct the CNN-GRU model;
3. Use the validation set to verify the model accuracy and save the model with the required accuracy;
4. Test the CNN-GRU model with the test set to obtain the final temperature prediction results on the cutting area of the tool.

As the original data set milling temperature and machining parameters and other types of data have different scales and value ranges, which makes some features weight update process will be affected by the larger and ignore some other features, normalization can eliminate this effect so that all features have the same scale. In addition, in this case, the difference in the scale of the features will affect the training and convergence of the model, if there is a large difference in the scale of the features, then the step size of the update in the gradient descent process may be affected by the difference in the size of the gradient, which will lead to a slower convergence speed. By normalization, the direction of gradient descent can be made consistent, accelerating the convergence speed of the model. There are many methods of normalization such as Min-Max Scaling, Z-score Standardization, Softmax Normalization etc. According to the data type choose the Min-Max Scaling method for data normalization, which is a common normalization method to scale the data to between [−1,1], the formula is shown as Equation (1):


(1) $$x^{*}=\frac{x_{i}-x_{\text{min}}}{x_{\text{max}}-x_{\text{min}}}$$

where x* represents the normalized data, *x_i_* represents the observed value at moment i, and *x_min_* and *x_max_* are the minimum and maximum values in the data, respectively.

The predictions of the model on the test set are restored after the model training using inverse normalization, which is formulated as Equation (2):


(2) $$x=x^{*}\left(x_{\text{max}}-x_{\text{min}}\right)+x_{\text{min}}$$

where *x* inverse normalized value, x* normalized value of the prediction result, and, *x_min_*, *x_max_* are the minimum and maximum values in the data, respectively.

To determine the model structure, this study employs Mean Squared Error (MSE) and R-squared (R^2^) as evaluation metrics. The formulas for these metrics are as Equations (3, 4):


(3) $$MSE=\frac{1}{n}\sum_{i=1}^{n}{\left({\hat{y}}_{i}-y_{i}\right)}^{2}$$

where *n* represents the total number of samples, *i* denotes the current sample, *ŷ_i_* is the predicted value for the *i*th sample, and *y_i_* is the true value for the *i*th sample. A smaller MSE indicates that the model’s predictions are closer to the true values, signifying better model performance.


(4) $$R^{2}=1-\frac{\sum_{i=1}^{n}{\left(y_{i}-{\hat{y}}_{i}\right)}^{2}}{\sum_{i=1}^{n}{\left(y_{i}-{\bar{y}}_{i}\right)}^{2}}$$

Where *n* represents the total number of samples, *i* denotes the current sample, *ŷ_i_* is the predicted value for the *i*th sample, *y_i_* is the true value for the *i*th sample, *ȳ_i_* represents the mean of the true values *y_i_*. The range of R^2^ is [0,1], with a higher R^2^ indicating better model performance.

In the inverse heat transfer problem solving model based on deep learning, effective data samples are the key to develop the model to accurately predict the boundary temperature conditions in the cutting region, among the 27 sets of full factorial test samples, the 24th set of test data is extracted as the test set data, and 80% of the remaining data is treated as the training set, and 20% is treated as the validation set. The machining parameters and the temperature at multiple points on the back face of the milling cutter are chosen as input features, and the temperature boundary conditions on the milling cutter cutting region are used as prediction labels. The compiled language for the neural network is Python 3.7, the model is built using the PyTorch deep learning framework, the operating system is 64-bit Windows 10, and the GPU is an NVIDIA GTX 1050Ti graphics card.

**Figure 4:**
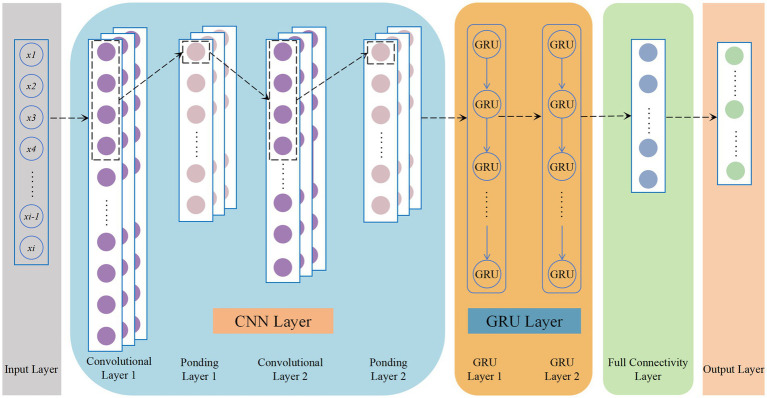
General structure of CNN-GRU model.

Among them, the parameters of the model are shown in Table 1, and the overall structure of the CNN-GRU model built in this paper is shown in Figure 4.

**Table 1:** CNN-GRU model parameters.

Model parameters	Numerical values
CNN Layers	2
CNN convolutional kernel size	4
Number of Convolutional Kernels	First layer 132, second layer 164
Number of GRU layers	2
Number of GRU neural units	First layer 50, second layer 50
Total number of neurons in the model	117,157

In order to confirm the validity and accuracy of the models, this paper compares the constructed CNN-GRU models with CNN, GRU, and LSTM networks, using MSE as the Loss function and R^2^ as the evaluation index of model error. All models use the dataset delineated in the previous section, and the parameter details of each model are shown in Table 2, and the training Epoch and batch size of all models are kept the same in order to ensure the scientific nature of model comparison.

**Figure 5:**
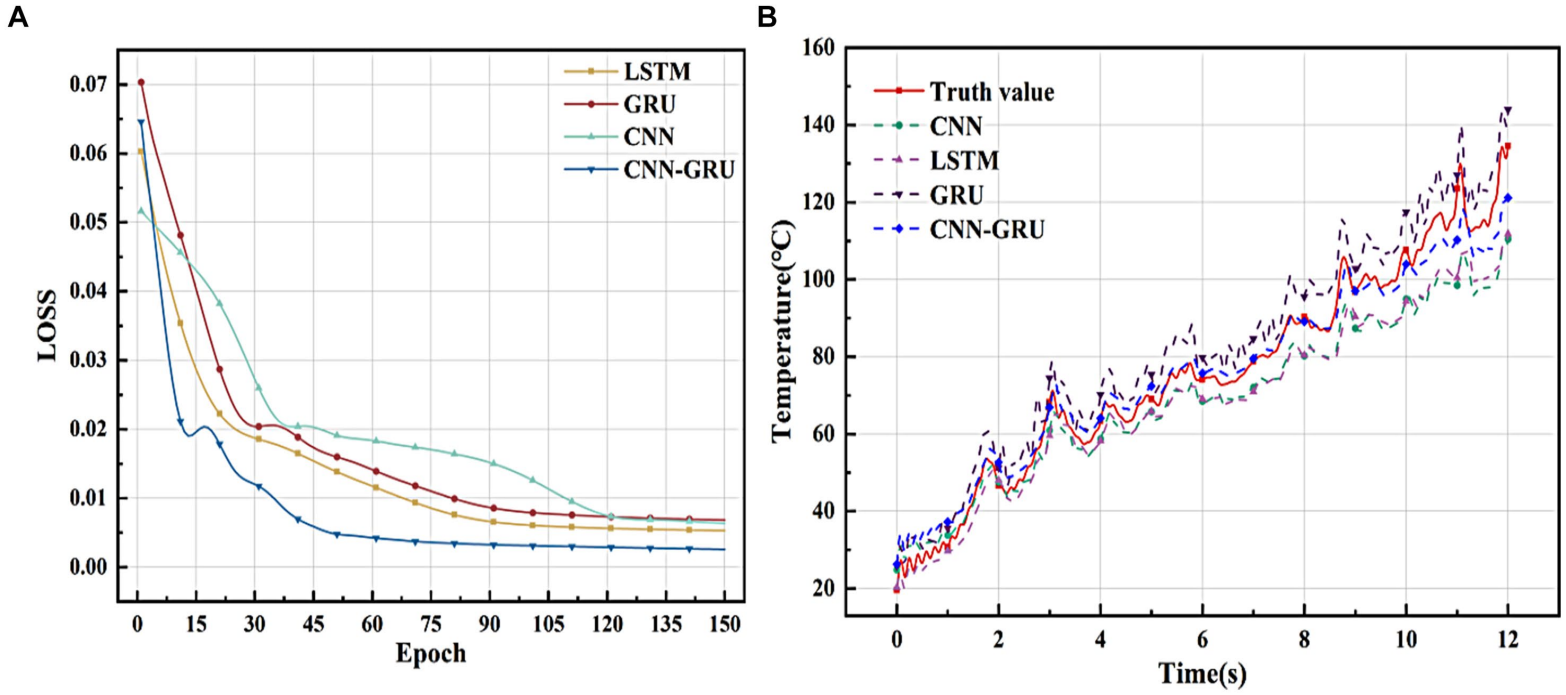
Performance comparison of different models. **(A)** The Loss function curves for each model training process. **(B)** Fit curves for each model on the test set.

The Loss function curves for the training process of each model are shown in Figure 5A. The figure illustrates that, for the same number of training iterations (150), the CNN model stabilizes its Loss value at approximately the 120th iteration, making it the slowest to converge among the models. In contrast, the LSTM and GRU models stabilize around the 90th iteration. Notably, the CNN-GRU model exhibits a smooth trend and stabilizes as early as the 45th iteration, demonstrating the fastest convergence speed among all the models.

**Table 2:** Parameter details for each model.

Network model	Epoch	Batch size	Total number of neurons in the model	Single training time(s)
LSTM	150	50	65,389	3.72
GRU	150	50	53,283	1.67
CNN	150	50	78,633	4.03
CNN-GRU	150	50	117,157	6.8

During 150 training sessions, the final LOSS value of the CNN-GRU model is 2.57 × 10^−3^, the final LOSS value of the CNN model is 6.37 × 10^−3^, the final LOSS value of the LSTM model is 5.3 × 10^−3^, and the final LOSS value of the GRU model is 6.82 × 10^−3^. In comparison, the CNN-GRU model exhibits better learning ability and fitting effect, and the evaluation indexes of each model are shown in Table 3.

**Table 3:** Evaluation indicators for each model.

Model	MSE	R^2^
CNN	6.37 × 10^−3^	0.89
LSTM	5.3 × 10^−3^	0.93
GRU	6.82 × 10^−3^	0.88
CNN-GRU	2.57 × 10^−3^	0.98

The fitting curves of each model on the test set are shown in Figure 5B, from which it can be seen that the prediction curves of the CNN-GRU model are the closest to the real value, and compared with other models, it can predict the temperature trend on the cutting area of the tool more efficiently, especially in the position of the peaks and valleys of the best fitting, which further verifies that the prediction results of the CNN-GRU model are more in line with the practical requirements.

### 3.3 Temperature boundary condition estimation model based on knowledge distillation with gated convolutional recurrent networks

**Figure 6:**
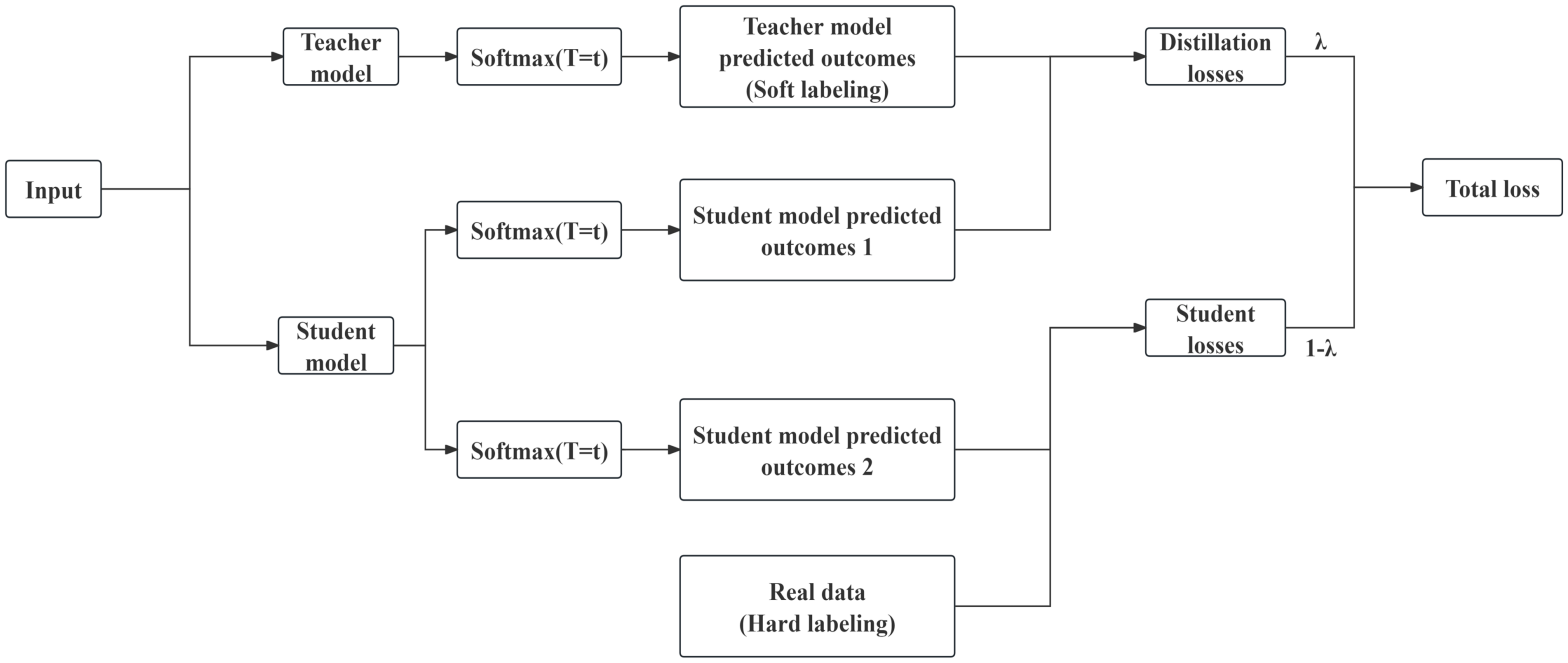
Structure of knowledge distillation strategy.

Knowledge distillation is an instructor-student training structure that typically utilizes a student model with a simpler network structure to learn the knowledge provided by an instructor model that has been trained with a more complex network structure; this approach trades a slight performance loss for faster computation and smaller model parameters. Knowledge distillation works by training the student model with both the predictions of the teacher model (soft labeling) and the real data (hard labeling), and calculating the weighted total loss of the student model on both the soft and hard labels, essentially “migrating” the knowledge learned by the teacher model to the student model. The structure of the knowledge distillation strategy used in this paper is shown in Figure 6.

The specific knowledge distillation strategy process is as follows:

1. The raw data that has been preprocessed is input to both the teacher model and the student model, the teacher model is the CNN-GRU model constructed in the previous section, and the student model is a small model with a single CNN layer and a single GRU layer.
2. The output of the teacher model is softened using the Softmax function with temperature coefficient T. The processed labels are used as soft labels.
3. Use the same Softmax function with temperature coefficient T to soften the results of the student model output, and process the labels of the student output and the soft labels of the teacher model output in the previous step through the distillation loss function LOSS_soft_ to obtain the distillation loss function between the student model and the teacher model.
4. Process the unsoftened student model output labels with the real hard labels through the student loss function LOSS_hard_ to get the student loss.
5. The distillation loss and the student loss are weighted to obtain the total loss, and the gradient of each parameter is updated in the backpropagation process.

The following are the calculation formulas involved in the knowledge distillation operation process:

Knowledge distillation soft labeling calculation formula as Equation (5):


(5) qi=expziT∑j=0kexpzjT

where T is the distillation temperature coefficient, used to control the “hardness” of the soft label. When T is larger, the soft label distribution area is uniform, more softened, when T is smaller, the soft label distribution closer to the hard label.

Distillation loss of the loss function LOSSsoft formula is as Equation (6):


(6) $$LOSS_{\mathrm{soft}}=\overset{k}{\underset{i=0}{\sum }}-p_{i}\left(u_{i},T\right)\log\left(p_{i}\left(z_{i},T\right)\right)$$

where *k* is the total number of samples, *p_i_*(*u_i_*,*T*) is the ith output of the teacher model at temperature coefficient T, and *p_i_*(*z_i_*,*T*) is the ith output of the student model at temperature coefficient T.

The loss function LOSShard for student loss is formulated as Equation (7):


(7) $$LOSS_{\mathrm{hard}}=\overset{k}{\underset{i=0}{\sum }}-y_{i}\log\left(p_{i}\left(z_{i},1\right)\right)$$

where *y_i_*7is a vector of hard labels representing the class i output of the unsoftened student model.

The total loss of knowledge distillation can be expressed as Equation (8):


(8) $$LOSS_{\mathrm{total}}=\mathrm{\lambda LOSS}+\mathrm{soft}\left(1-\lambda \right)LOSS_{\mathrm{hard}}$$

where *λ* are hyperparameters, which are fixed constants that can be empirically tuned to the reference or dynamically adjusted.

**Figure 7:**
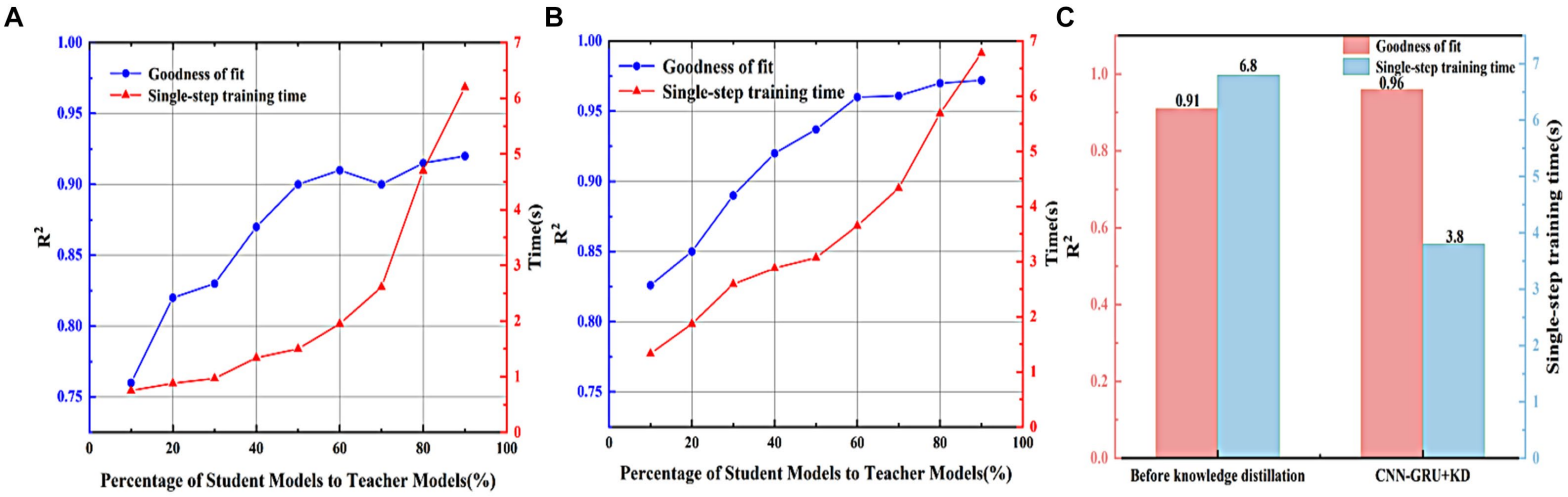
Comparison of model performance before and after adding knowledge distillation. **(A)** Comparison of student model training results for each scale. **(B)** Comparison of student model predictions for each scale after knowledge distillation. **(C)** Comparison of model performance improvement before and after acceleration of knowledge distillation strategy.

Based on the above knowledge distillation strategy for model optimization design of the constructed CNN-GRU teacher model, the first step is to construct a simple CNN-GRU student model, and with reference to the structure of the teacher model with 2 layers of CNN layers plus 2 layers of GRU layers, the student model structure is designed as a 1-layer CNN layer plus 1 layer of GRU layer structure. In order to determine the optimal student model total neuron number, the student models with total neuron number of 10, 20, 30, 40, 50, 60, 70, 80, and 90% of the teacher’s model were designed, and the gradient descent training was performed on each model using the same training, validation, and test sets, and the training Epoch and batch sizes were consistent with those of the teacher’s model. The learning ability and single-step training time of each student model not trained by the knowledge distillation strategy are first compared to the true values, and the comparison of the prediction results of each percentage of student models is shown in Figure 7A.

As can be seen from the figure, the goodness of fit of the student model gradually increases with the increase of the total number of neurons before the knowledge guidance of the teacher’s model, and the goodness of fit tends to stabilize when the ratio of the student model to the teacher’s model is 60%, which indicates that the closer the student model is to the teacher’s model, the better the ability to learn the data, however, due to the structural limitation of the student model, the simple model structure is not enough to accurately reflect the complex nonlinear relationship between the input data and the output data. However, due to the structural limitations of the student model, the simple model structure is not enough to accurately reflect the complex nonlinear relationship between the input data and the output data, although the goodness of fit of the student model to the teacher’s model still fluctuates slightly after the ratio of the student model to the teacher’s model is more than 60%, but the overall learning ability does not improve much. The single-step training time consumed by the student model also becomes more with the increase of the total number of neurons, and the rate of change of the single-step training time consumed increases when the ratio of the student model to the teacher’s model is 70%, which demonstrates that the closer the number of neurons of the student model is to that of the teacher’s model, the slower the model’s inference is, and the more hardware resources it occupies.

Then, using the knowledge distillation strategy, the teacher model trained in the previous section is used to “guide the training” of the above student models of different sizes, so as to transfer the knowledge learned from the teacher model to the student model. In order to avoid random errors, the distillation temperature coefficient T is set to [1,10], T takes an integer, the total loss weighting factor *λ* is set to [0.1,0.9], *λ* retains one decimal place, and the distillation effect of the model under the parameter combinations of T and *λ* is compared one by one, and it is finally determined that T = 7, *λ* = 0.8. The comparison of the prediction results of various proportions of the students’ models after the distillation is shown in Figure 7B.

As can be seen from the figure, the student models (CNN-GRU + KD) guided by the teacher’s model all have a better improvement in the goodness-of-fit, and the R^2^ shows a smooth trend and stabilizes around 0.96 when the percentage of the student model to the teacher’s model is 60%, which results in a longer single-step training time than that of the original model after the distillation before the original model is longer, when the percentage is more than 80%, the single-step training time of the model is close to that of the teacher model. Therefore, considering the goodness of fit of the student model and the single-step training time, the total number of neurons of the student model is determined to be 60% of the teacher model, and the network parameters of the student model are shown in Table 4. A comparison of the performance improvement of the model before and after acceleration by the knowledge distillation strategy is shown in Figure 7C.

**Table 4:** Network parameters of the student model.

Model parameter	Numerical values
CNN Layers	1
CNN convolutional kernel size	4
Number of convolution kernels	148
GRU layers	1
Number of GRU neural units	90
Total number of neurons in the model	70,170

From Figure 7C, it can be seen that the CNN-GRU + KD model accelerated based on the knowledge distillation strategy has an accuracy of 0.96, which compares with the teacher’s model although there is some performance loss (loss of 2%), but it improves the prediction accuracy by 5% over the student’s model of 0.91, and the training time of the CNN-GRU + KD as usual reduces by 44.1% compared with the teacher’s model, which proves that the acceleration of the knowledge distillation strategy is feasibility of the compression model.

## 4 Experimental result and analysis

### 4.1 Model noise resistance test

In 3.3, the model accuracy and computation time have been discussed, and the results show that he CNN-GRU+KD model achieves significant time acceleration with minimal loss in accuracy compared to the teacher model, making it more suitable for the rapid provision of necessary data for fast temperature field reconstruction.

**Figure 8:**
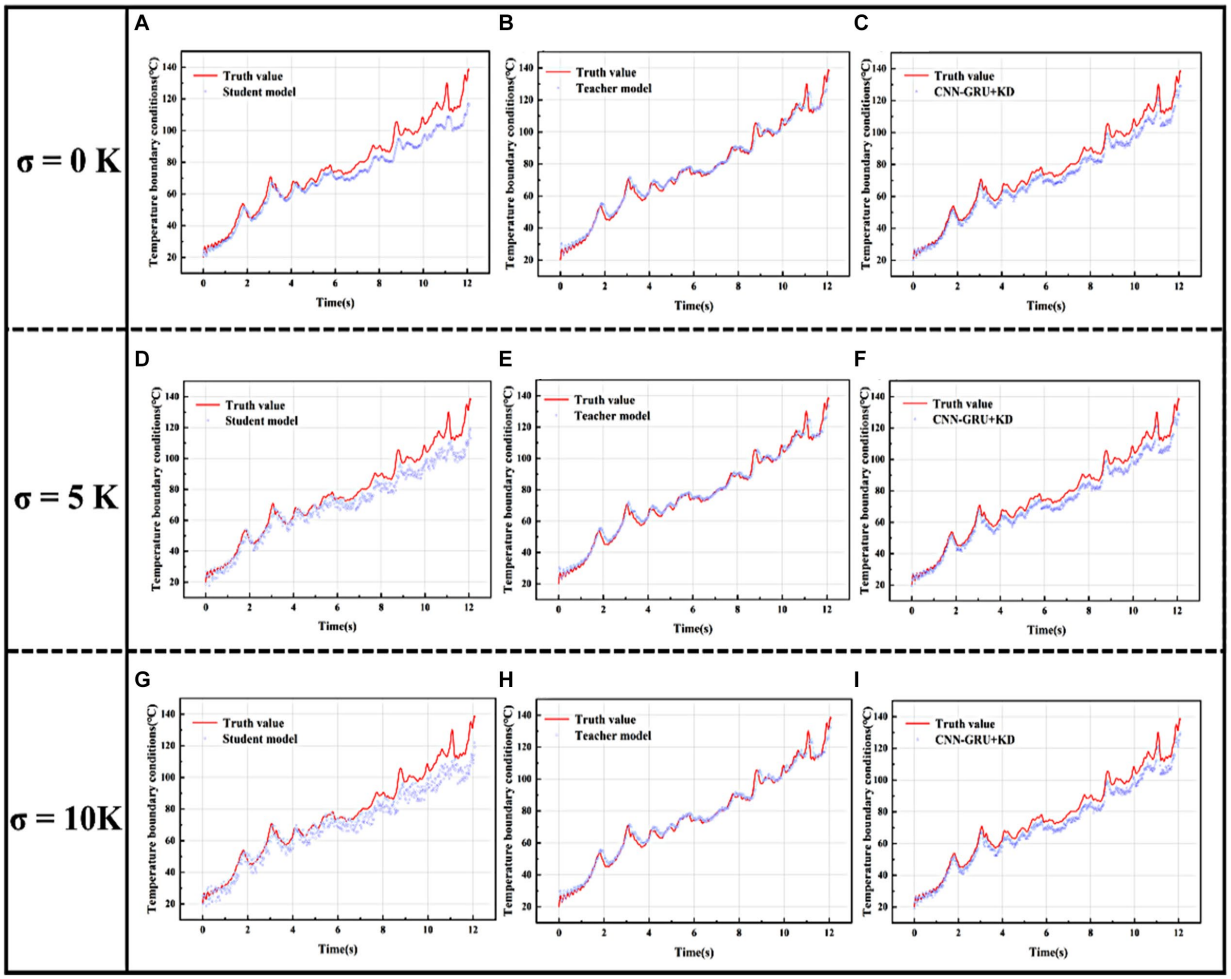
Test set fitting curves for the three models at different noise levels. **(A)** Noise level σ=0K student model fit curve on test set. **(B)** Noise level σ=0K teacher model fit curve on test set. **(C)** Noise level σ=0K CNN-GRU + KD model fit curve on test set. **(D)** Noise level σ=5K student model fit curve on test set. **(E)** Noise level σ=5K teacher model fit curve on test set. **(F)** Noise level σ=5K CNN-GRU + KD model fit curve on test set. **(G)** Noise level σ=10K student model fit curve on test set. **(H)** Noise level σ=10K teacher model fit curve on test set. **(I)** Noise level σ=10K CNN-GRU + KD model fit curve on test set.

In addition to model accuracy and computation time, the model’s noise immunity to data noise is especially critical in the solution of the inverse heat conduction problem, because the inverse heat conduction problem is essentially an “unsettled” problem, which is more sensitive to the noise of the signal, and therefore the model’s noise immunity needs to be tested. The CNN-GRU + KD after knowledge distillation is tested with the teacher model and the student model on the dataset with noise level σ=0K , noise level σ=10K , and noise level σ=10K , respectively, and the results are shown in Figures 8A–I. Table 5 shows the MSE computed by the student model, the teacher model, and the CNN-GRU + KD Model on the test set under the noise level of these three sets of data.

**Table 5:** MSE of the three models at different data noise levels.

Noise level	Model		CNN-GRU + KD	Student model	Teacher model
σ=0K	6.42 × 10^−3^	2.57 × 10^−3^	2.85 × 10^−3^
σ=5K	7.89 × 10^−3^	3.02 × 10^−3^	3.52 × 10^−3^
σ=10K	1.69 × 10^−2^	3.95 × 10^−3^	4.47 × 10^−3^

As can be seen from the learning effects of the above three models at different noise levels, the student model with fewer model parameters, simple structure, and no knowledge distillation training performs the worst as the noise level increases, and has been severely distorted at noise level σ=10K . The teacher model, on the other hand, can still learn the trend of real data in noisy data due to its complex model structure and more model parameters, and shows better noise immunity, which can be attributed to the dimensionality reduction of the noisy data by multilayer convolutional pooling, thus compressing the random noise information. Thus the CNN-GRU + KD trained based on the teacher model learns the better noise immunity of the teacher model and shows better robustness compared to the student model, and the stability of the CNN-GRU + KD is still satisfactory even in the case of high noise level.

### 4.2 Simulation reconstruction

**Figure 9:**
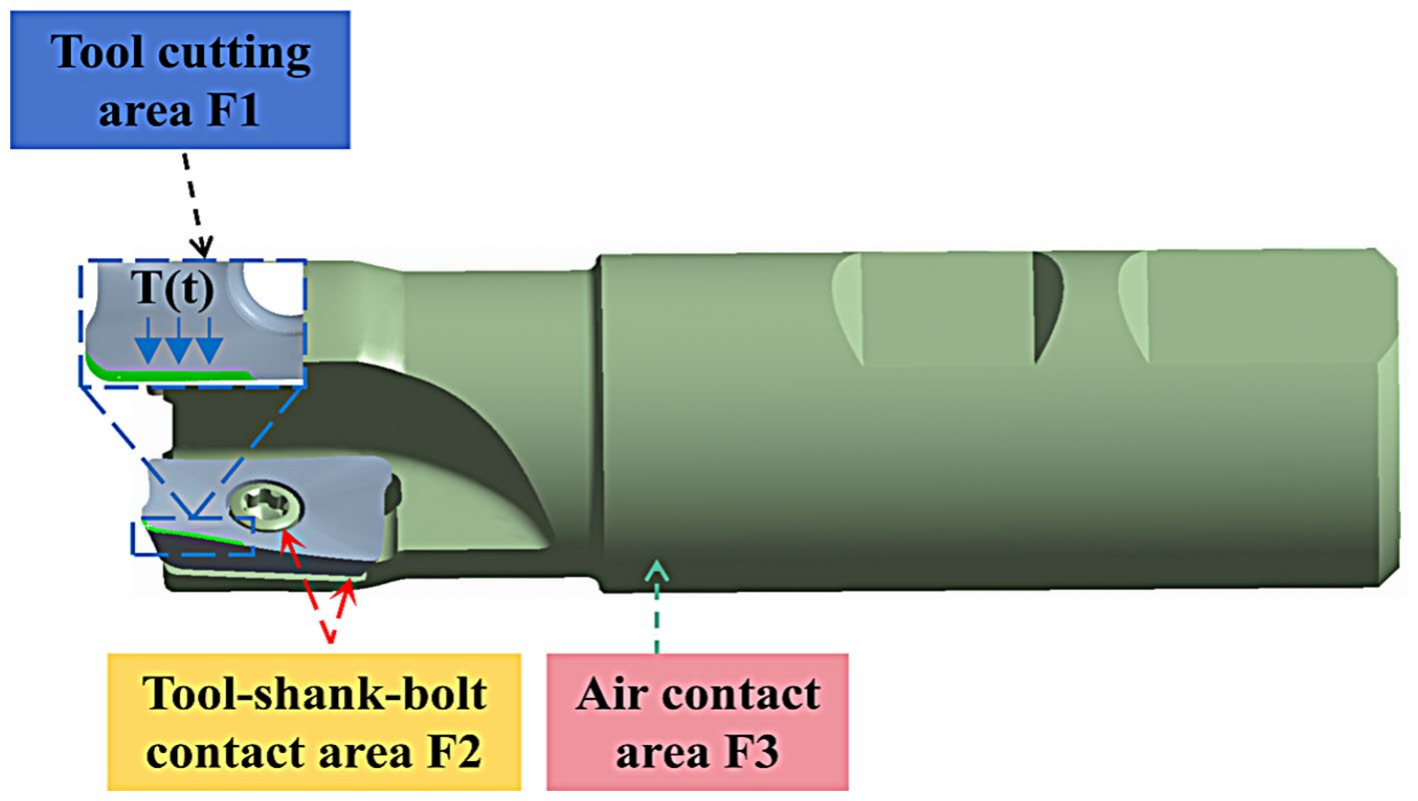
Three-dimensional schematic diagram of the tool-shaft-bolt cutting system.

In the milling process, the heat in the milling cutter is mainly transferred in three ways: the mutual transfer of heat between the tool-workpiece-chips in the cutting area, the convective heat transfer between the tool-stem-air, and the thermal radiation heat transfer in the high temperature region, because the radiation area of the high temperature region in the process of metal cutting is generally very small, so the thermal radiation heat transfer is generally negligible. Therefore, before constructing the transient heat transfer model of double-edged milling cutter, if only simplified two-dimensional or three-dimensional modeling for the milling cutter will greatly affect the accuracy of the heat transfer model, should be three-dimensional modeling of the cutting system as a whole, including inserts, shanks, bolts, etc. This paper in accordance with the actual tool as a whole to establish a three-dimensional geometric model shown in Figure 9, and in accordance with Figure 1 in the cutting area of the tool cutting region image of the model tool cutting area location for detailed division.

The tool cutting area is defined as F1, the tool-stem-bolt contact area is defined as F2, and the contact area between the cutting system and the air is defined as F3. The heat flow inside the system during the whole milling process can be understood as follows: the cutting heat enters into the overall cutting system from the tool cutting area, and then transfers to the tool stem and bolt through the tool-stem-bolt contact area, and the heat dissipation is realized by convective heat transfer with the surrounding air in the contact area of the cutting system and the air. The cutting system in contact with the air through the convective heat transfer with the surrounding air to achieve heat dissipation, this process is a complex, three-dimensional, transient heat transfer process, the control equation of heat transfer can be expressed as Equation (9):


(9) $$\frac{\partial }{\partial x}\left(k_{t}\frac{\partial T}{\partial x}\right)+\frac{\partial }{\partial y}\left(k_{t}\frac{\partial T}{\partial y}\right)+\frac{\partial }{\partial z}\left(k_{t}\frac{\partial T}{\partial z}\right)=\rho c_{t}\frac{\partial T}{\partial t},t>0$$

where T represents the transient temperature at the internal point of the tool heat transfer model, (*x,y,z,t*) are the spatial and temporal variables of the tool heat transfer model, and *k_t_* and *c_t_* represent the specific heat capacity and thermal conductivity of the material and ρ the material density.

**Figure 10:**
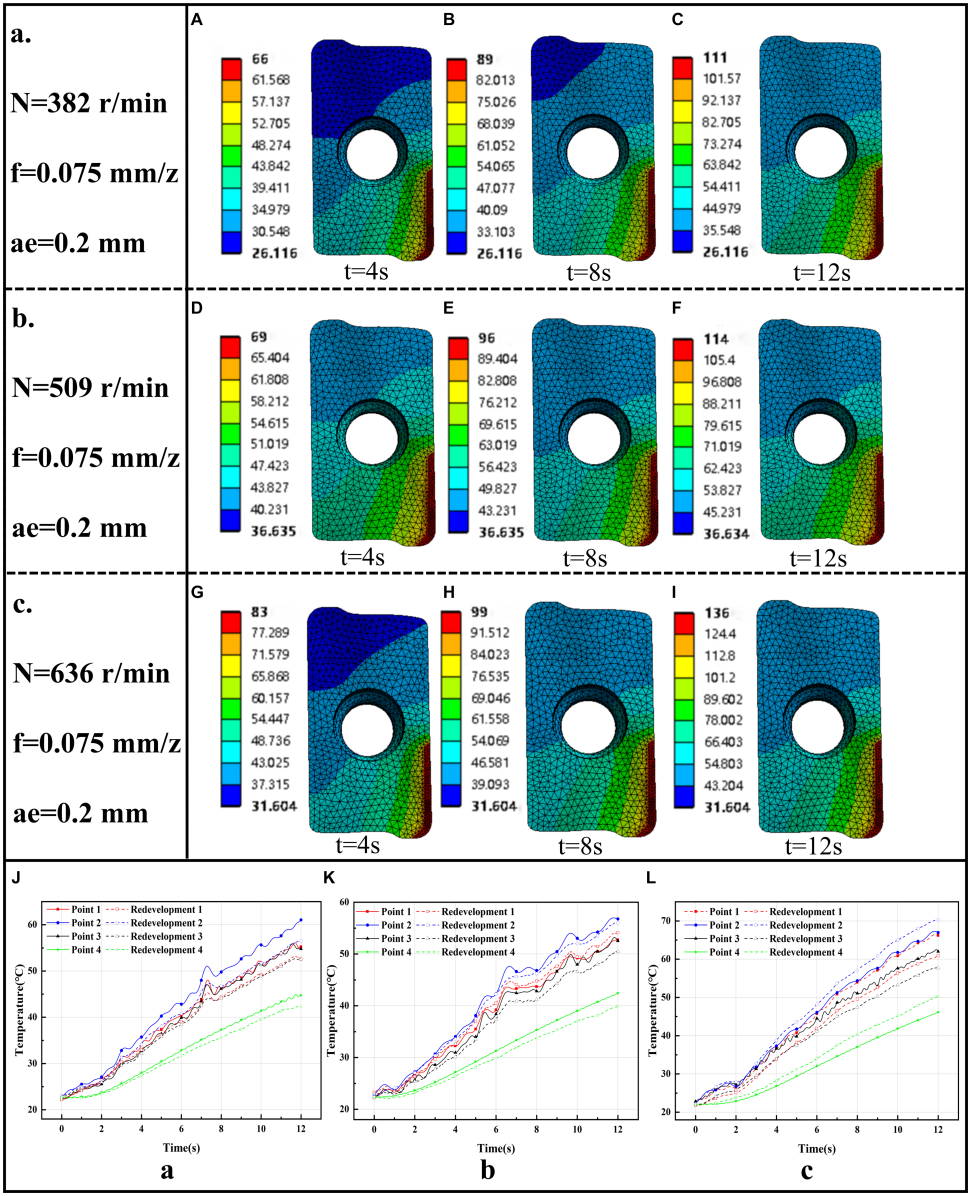
Temperature field reconstruction results for three different operating conditions. a. Tool Temperature Field Reconstruction Results for Case *N* = 382 r/min, *f* = 0.075 mm/z, ae = 0.2 mm. **(A)** t = 4 s. **(B)** t = 8 s. **(C)** t = 12 s. b. Tool Temperature Field Reconstruction Results for Case N = 509 r/min, f = 0.075 mm/z, ae = 0.2 mm. **(D)** t = 4 s. **(E)** t = 8 s. **(F)** t = 12 s. c. Tool Temperature Field Reconstruction Results for Case *N* = 636 r/min, f = 0.075 mm/z, ae = 0.2 mm. **(G)** t = 4 s. **(H)** t = 8 s. **(I)** t = 12 s. **(J)** *N* = 382 r/min, f = 0.075 mm/z, ae = 0.2 mm Reconstruction temperature vs. measured temperature. **(K)** *N* = 509 r/min, f = 0.075 mm/z, ae = 0.2 mm Reconstruction temperature vs. measured temperature. **(L)** *N* = 636 r/min, f = 0.075 mm/z, ae = 0.2 mm Reconstruction temperature vs. measured temperature.

Based on the proposed CNN-GRU + KD model, the actual measured temperature of the back face of the milling cutter and the machining parameters are inputted into the model to predict the temperature boundary value on the cutting area of the milling cutter, and the estimated temperature boundary conditions are inputted into the transient heat transfer model of the milling cutter to realize the reconstruction of the tool temperature field during the milling process. In this paper, the tool temperature field reconstruction is carried out for different rotational speeds under the feed rate *f* = 0.075 mm/z per tooth, radial depth of cut (ae) of 0.2 mm, and the milling mode is dry cutting and reverse milling. According to the temperature boundary conditions prediction results and double-edged milling cutter transient heat conduction model combination of the milling process tool temperature field reconstruction, respectively, take 4 s, 8 s, 12 s to draw the temperature field of the pre-milling, mid-term, the end of the milling period, the results are shown in Figures 10A–I.

In order to verify the accuracy of the milling process tool temperature field reconstruction model, the milling cutter transient heat transfer finite element calculation results corresponding to the location of the thin-film thermocouple sensor temperature measurement point of the unit temperature reconstruction curve exported and compared with the sensor’s actual measurement of the milling temperature curve, the results of the reconstruction of the temperature and the measured temperature comparison results in different working conditions are shown in Figures 10J–L.

As can be seen from Figures 10A–I, in the pre-milling process, the tool cutting area produces a large amount of cutting heat, which will spread around to form a temperature gradient, with the cutting process heat generation and dissipation to reach an equilibrium state, the tool near the cutting area near the temperature distribution gradually tends to stabilize, while the tool distal space on the tool is still part of the region’s temperature field distribution in the change, because this part of the is farther from the cutting region, the heat conduction is slower, and it takes longer to reach the thermal steady state. In addition, it can be seen that there is a large temperature gradient at the tip of the milling cutter, the temperature gradient distribution on the front face is very uneven, and the high temperature region is mainly concentrated in the vicinity of the cutting area and the temperature increases with time.

From Figures 10J–L, it can be seen that the reconstructed temperature curve of the milling process fits well with the temperature curves of the four temperature measurement points. Subject to the actual measurement temperature of the sensor, the fit between the reconstructed temperature curves of the four temperature measurement points at the locations of the above three conditions and the actual measurement temperature curves of the sensor is as high as 0.97, and the fit is as low as 0.92, with the fit above 0.9, which proves that there is a good fitting relationship between the reconstructed temperature curve There is a good fitting relationship between the reconstructed temperature curve and the actual measured temperature curve of the sensor. The Root Mean Square Error (RMSE) was chosen as the evaluation index for calculating the error between the reconstructed temperature profile and the actual measured temperature profile, and the formula of RMSE is as Equation (10):


(10) $$\mathrm{RMSE}=\sqrt{\frac{\sum_{i=1}^{n}{\left(y_{i}-{\hat{y}}_{i}\right)}^{2}}{n}}$$

where n represents the data points, σ=10K represents the measured temperature value of the sensor at time i moment, and ${\hat{y}}_{i}$ represents the modeled temperature result at time i moment.

The RMSE between the reconstructed temperature curve at the temperature measurement point when the spindle speed *N* = 509 r/min and the actual temperature curve measured by the sensor is 1.85, 1.98, 1.62, and 1.43°C; the RMSE between the reconstructed temperature curve at the temperature measurement point when the spindle speed *N* = 636 r/min and the actual temperature curve measured by the sensor is 4.51, 2.89, 3.68, and 3.56°C, which is in the acceptable range. The above calculations prove the accuracy and feasibility of the tool temperature field reconstruction method for milling process based on the inverse heat conduction problem used in this paper.

## 5 Conclusion

In this paper, a solution model for the inverse heat transfer problem based on a convolutional gated recurrent network to predict the temperature boundary conditions in the cutting region of the tool is proposed. And the CNN-GRU model that will be built is compressed and accelerated using the knowledge distillation strategy, and the big model that will be built is considered as the teacher model, and the small CNN-GRU is designed, which is considered as the student model. The goodness-of-fit of the CNN-GRU + KD model trained by the teacher model guidance is 0.96, and the single-step training time of the model is reduced by 44.1% compared to the teacher model. Compared to than the student model without teacher model guidance, the accuracy of the CNN-GRU + KD model increased by 5% and the mean square error decreased by 55%. By adding different levels of random noise to the model input data, the CNN-GRU + KD model learns the noise-resistant ability of the teacher model and still shows good robustness and stability under noisy data.

The transient heat transfer model of the tool is constructed, and all the surface areas of the model are divided into three major areas, namely, the tool cutting area F1, the tool-shank-bolt contact area F2, and the contact area between the cutting system and the air F3, according to the actual situation, and the boundary conditions on each area are defined according to the theory of heat transfer. Based on the CNN-GRU + KD model predicted temperature boundary conditions in the tool cutting region, combined with the tool transient heat conduction model, the temperature field of the milling cutter was reconstructed for three different working conditions, and the reconstructed temperature curves of the milling process at the location of the temperature measurement points and the temperature curves of the sensors were calculated for the goodness-of-fit and the root-mean-square error, and the goodness-of-fit of curves in the three working conditions was the highest of 0.97. The minimum root mean square error is 1.43°C, which are in the acceptable range, and the reconstruction of tool temperature field in milling process is realized.

Based on the results of this paper, future research could further enhance the CNN-GRU+KD model for predicting temperature boundary conditions in the tool cutting region. One promising direction is to integrating intuitionistic fuzzy approaches, as demonstrated by Versaci and La Foresta (2024) in energy management. Fuzzy systems could more effectively handle the uncertainty and noise inherent in temperature data. By integrating fuzzy rules based on operator experience, the CNN-GRU model could become more versatile and adaptable to various milling scenarios, thereby improving the robustness and adaptability of the CNN-GRU+KD model under different operating conditions. Combining intuitionistic fuzzy approaches with the CNN-GRU+KD model could lead to even greater performance improvements, particularly in situations where real-time decision-making and noise immunity are critical.

## Data Availability

The data that support the finding of this study are available from the corresponding author upon reasonable request.
